# Video Q&A: Excess body weight and cancer

**DOI:** 10.1186/1741-7015-11-2

**Published:** 2013-01-03

**Authors:** Rudolf Kaaks

**Affiliations:** 1 Division of Cancer Epidemiology, German Cancer Research Center (DKFZ), 69120 Heidelberg, Germany

## Rudolf Kaaks talks on Excess body weight and cancer

## Introduction

Rudolf Kaaks originally trained at Wageningen University in the Netherlands, and is currently Professor of Cancer Epidemiology and head of the Division of Cancer Epidemiology at the German Cancer Research Center (DKFZ). He is also one of the principal investigators of the European Investigation on Nutrition and Cancer (EPIC). His research focus is on metabolic and endocrine pathways that may provide a link between lifestyle, nutritional energy balance and cancer risk, and he has conducted various epidemiologic studies in prospective cohorts to examine cancer risks in relation to blood levels of endogenous hormones, growth factors, inflammation factors and other metabolic factors. Furthermore, he has a strong interest in linking findings from epidemiologic studies on energy balance and cancer with those from basic physiology, clinical sciences and molecular biology.

## Edited transcript

### 1) What is excess body weight?

Excess body weight is a term often used to indicate a person is heavier than they should be. As people become fatter they can lose muscle mass, but gain body weight in terms of excess fat tissue. This fat tissue may deposit in different parts of the body, such as under the skin as subcutaneous fat or around the abdomen as intra-abdominal fat. Excess body weight as a general term is associated with adverse health outcomes linked to adiposity. Fat deposited on the hips, which is more prevalent in women, carries fewer health risks than fat in the abdomen and the subcutaneous region of the abdomen. So, it is particularly the latter two components that are actually targeted by the term.

### 2) How are obesity and overweight categories defined?

Obesity and overweight categories are defined by the World Health Organization (WHO) in terms of body mass index (BMI). Body mass index (which is weight divided by height to the square), is an index that gives a global impression of how large someone is for a given size in terms of height. This correlates strongly with body fatness, and the overweight category is classified by a BMI of 25 to 30, while obesity is classified as a BMI of 30+. The latter two categories combined are also called excess body weight categories - but that is a more pragmatic interpretation of the term because these categories are defined by the WHO and do not always match the limits at which adverse health consequences become apparent.

### 3) Which particular types of cancers are linked to excess weight?

Excess weight is a clear risk factor for a number of cancers that are becoming prevalent in industrialized parts of the world. In women, cancer of the endometrium and breast are particularly prevalent, and excess weight and adiposity also increases the risk of breast cancer after menopause. In men and women, colon cancer, renal cancer and adenocarcinoma of the esophagus is strongly associated with obesity. Aggressive prostate cancer risks are clearly increased in men that are in the overweight or obese categories.

The relative risks associated with excess weight vary - the strongest associations are seen in adenocarcinoma of the esophagus and in endometrial cancer where subjects in the obese category may have a 3.5- to 4-fold greater risk compared to those in the lean category. For some other cancers, these relative risks can be less, but from an epidemiological perspective, there is a strong connection between excess weight and cancer risk for a large number of tumor types.

### 4) Is there any evidence to suggest that exercise and calorie restriction can protect against cancer development?

Yes, there is. There have been numerous epidemiological studies where data have been collected, mainly via questionnaires, on levels of physical activity. Almost invariably, these studies show that those who have higher levels of physical activity tend to have lower risks of colon cancer. There is also substantial evidence that indicates a reduced risk of breast cancer. These are two of the more frequent cancer types that have, therefore, also been studied more intensely, and it is quite possible that for further cancer types there are quite similar inverse relationships. This has also certainly been documented in other tumor types, but globally, the evidence is not yet as strong as it is for colon and breast cancer.

### 5) What are the mechanistic insights?

The two factors that jointly contribute to alterations in hormone metabolism occur when people become overweight and remain physically inactive.

A first, very important hormonal metabolic change of obesity associated with physical inactivity is insulin resistance. This is a lack of response of muscle, liver and other tissues to insulin in terms of glucose uptake from the blood, and is compensated for by an increase in pancreatic insulin secretion, so people that have insulin resistance tend to have higher fasting and non-fasting insulin levels.

Insulin is well-known as a hormone, but is increasingly believed to also act as a growth factor, which can stimulate growth and proliferation of cells as well as inhibit the apoptosis of cells, and through these actions can favor the development of tumors.

Another set of hormones that are very important, particularly in relation to endometrial and breast cancer, are the sex hormones - especially estrogens. After menopause, women no longer produce estrogens in their ovaries, but they continue to produce androgens in the adrenal glands and in the ovaries. These androgens can be converted in fat tissue into estrogens, and it has been observed that as women gain weight after menopause, they also have higher circulating estrogen levels in the blood. These estrogens play an important role in the development of endometrial cancer. It is well known from a number of experimental, as well as epidemiological, observations that raising levels of estrogens in the blood will promote the development of both endometrial and breast tumors.

Another component that is a little bit more complicated but quite important with respect to endometrial cancers in particular, is that in pre-menopausal women, obesity is also strongly linked to a condition called ovarian hyperandrogenism - that is, the ovaries produce excess levels of androgens. This is also often referred to as polycystic ovary syndrome. We know that hyperinsulinemia plays an important role in the stimulation of androgen synthesis by the ovaries, and when this syndrome develops, we see chronic anovulation. Also, in pre-menopausal women, production of progesterone declines in the second phase of the menstrual cycle, and this absence of progesterone is also a strong contributing factor to the development of endometrial cancer.

Other hormones that are being implicated include the insulin-like growth factors. Higher levels of insulin lead to higher fractions of insulin-like growth factors that can reach target tissues in the sepsus, and there the free, biologically active fraction of IGF-1 is increased in the blood and other tissues of hyperinsulinemic subjects. In fact, insulin-like growth factors, which share a great deal of homology with insulin, also promote tumor development by promoting cell growth, division and, in particular, apoptosis.

### 6) If an obese person loses weight and reaches a normal BMI, does their risk of developing obesity-related cancers decrease to similar levels as non-obese people?

There is very little epidemiological evidence that suggests this might happen, but that is probably due to the fact there are not many previously obese people who manage to maintain a lower weight and BMI for a very long period of time. Therefore, it is hard to find sufficient numbers of people that fall within this category, who can be observed to measure how their risks of developing cancer may change in comparison to those who did not lose weight.

There is, however, strong physiological evidence from other sources to suggest that some hormonal effects that mediate the effects of adiposity on cancer development, particularly the late-stage growth promoting effects, can be influenced by weight loss of the individual. For example, weight loss in an obese woman can lead to lower estrogen levels, which might lead to lower cancer risk as a consequence. However, as I've explained before, as most people do not usually manage to lose weight and maintain that lower weight for a long enough period of time, research into this area is limited. So, a strong general recommendation that has come out of epidemiological research is to avoid weight gain throughout life, rather than try to revert to a normal weight once the ideal BMI has been exceeded.

### 7) What are the associations between age, adiposity and cancer development?

That is still, by and large, an open question. We currently do not have enough evidence to draw strong conclusions, but it is quite plausible that excess weight may lead to early stage effects of cancer - leading through hyperinsulinemia, for example, to early stage genetic alterations that may be the first steps to tumor development. It is plausible that accumulations of such early mutations may be favored by a hyperinsulinemic state for some tumors, but as I already mentioned it can also be the later stage effects on growth that may be linked to hormones, which are present at higher levels in obese people. So it might well be both, but for the moment this is more of a speculation, than something that we have really been able to prove in epidemiology.

It is very difficult to unconvolute certain elements in this type of research. We do know that persons who have higher degrees of excess weight and BMI tend to have a higher risk of developing cancer compared to those within the intermediate BMI range. One possible explanation is that this is not just due to a dose response effect in terms of the metabolic alterations, but also that people who are at the higher end of the obesity scale may have been obese for many years, because this is a condition that generally develops rather slowly.

My speculation is that with the obesity epidemic, which includes young people, we may start to observe an earlier occurrence of some tumors that are linked with obesity.

### 8) What does this mean in terms of the impact of obesity on public health and, therefore, on the drive to prevent obesity-related cancer ?

As I mentioned earlier, there is a relative increase in risk in obese people compared to leaner people. The relative risks vary from slightly below a two-fold increase to up to a four-fold increase, depending on the tumor type. Based on the relative risk and also on the prevalence of excess weight in a population, we can make estimates of the population attributable fraction (PAF) - that is, the part of overall cancer occurrence, which potentially relates to weight as a separate risk factor. When we do such calculations we clearly come to rather high estimates for a number of tumors that are frequent in our industrialized societies. The PAF may be 20% for colon cancer, as high as 50% for endometrial cancer, or 40% for renal cell tumors, to name but a few examples.

Overall, if all the cancer occurrences in a country such as the USA or the Western European countries are considered, it is quite clear that after smoking, excess weight is probably the most important factor that one should try to avoid in order to reduce the cancer burden - perhaps together with an increase in physical activity.

### 9) Where can I find out more?

See reference list: [1-5]

## Pre-publication history

The pre-publication history for this paper can be accessed here:

http://www.biomedcentral.com/1741-7015/11/2/prepub

## Supplementary Material

Rudolf Kaaks talks on Excess body weight and cancer Click here for file
